# Exploring the Neural Substrates of Number Sense: A Perspective on Genetics, Behaviour and Neural Circuity

**DOI:** 10.1111/ejn.70506

**Published:** 2026-04-21

**Authors:** Mirko Zanon, Caroline H. Brennan, Maria Elena Miletto Petrazzini, Jose V. Torres‐Pérez

**Affiliations:** ^1^ Center for Mind/Brain Sciences University of Trento Rovereto Italy; ^2^ School of Biological and Behavioural Sciences Queen Mary University of London London UK; ^3^ Department of Biomedical Sciences University of Padova Padova Italy; ^4^ Department of General Psychology University of Padova Padova Italy; ^5^ Departament de Biologia Cel·lular, Biologia Funcional i Antropologia Física Universitat de València València Spain

**Keywords:** approximate number system, numerosity, object tracking system, Williams syndrome, zebrafish

## Abstract

Number sense is the intuitive, non‐verbal ability to perceive and process numerical quantities without formal counting. This evolutionarily conserved trait, shared by different animal species, is supported by two mechanisms: the object tracking system (OTS) for small sets and the approximate number system (ANS) for larger quantities. Although historically viewed as distinct, these systems interact dynamically; their disruption is implicated in developmental dyscalculia and disorders such as Williams–Beuren syndrome (WBS), a chromosome microdeletion characterised by marked numerical and visuospatial deficits. Here, we synthesise neurobiological advances to provide an integrative perspective on the neural substrates of number sense. Field studies provide ecological validity, while laboratory procedures offer tighter precision; together, they illuminate the biological foundations of numerical cognition. Although number sense is conserved, human studies indicate only moderate heritability likely reflecting directional selection. Nonetheless, genetic findings converge on neurodevelopmental and synaptic mechanisms. Zebrafish ( *Danio rerio* ) offer a powerful platform to bridge genes, circuits and behaviour. For instance, manipulating zebrafish genes linked to WBS reveals gene‐specific effects on quantity processing. At the neural level, numerical cognition is largely supported by specialised number‐selective neurons. Whole‐brain calcium imaging in larval zebrafish demonstrates that these neurons emerge by 3 days postfertilisation and follow a trajectory where representations of small numerosities precede larger ones. Altogether, integrating genetic, behavioural and circuit‐level approaches provides a powerful framework for uncovering conserved mechanisms of numerosity supporting higher‐level cognitive functions, including mathematics.

## Graphical abstract

Numerical cognition arises from conserved mechanisms linking genes, neural circuits and behaviour. Using zebrafish as a tractable model, behavioural assays, whole‐brain imaging and genetic analyses can be integrated to identify neural and molecular bases of quantity discrimination. This framework provides a platform for studying numerical cognition and its disruption in neurodevelopmental disorders.

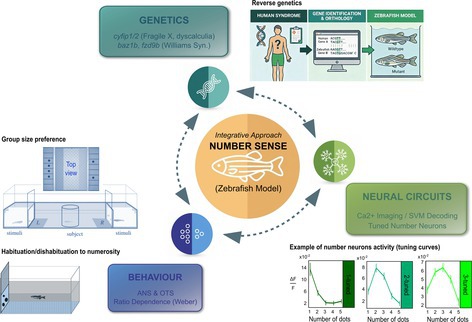

**Abbreviations**
AMPK: AMP‐activated protein kinase
ANS: approximate number system
ARK5: AMPK‐related protein kinase 5 (NUAK1)
BAF: BRG1/BRM‐associated factor (chromatin remodelling complex)
BP1–BP2: Breakpoints 1 and 2 of the 15q11.2 copy number variation region
CNV: copy number variation
CRISPR/Cas9: clustered regularly interspaced short palindromic repeats/CRISPR‐associated protein 9
dpf: days postfertilisation
ECM: extracellular matrix
eIF4E: eukaryotic translation initiation factor 4E
FMR1: Fragile X Mental Retardation 1
GABA: gamma‐aminobutyric acid
GSP: group size preference
GWAS: genome‐wide association study/studies
KAR: Kainate receptor
LoF: loss‐of‐function
OMIM: Online Mendelian Inheritance in Man
OTS: object tracking system
SVM: support vector machine
TGF‐β: transforming growth factor beta
WBS: Williams–Beuren syndrome
WT: wild‐type

## 1 Introduction

Numbers play a fundamental role in our everyday activities, such as scheduling appointments, managing money, using passwords and measuring ingredients while cooking. Behavioural and neuroimaging studies show that humans have two systems for processing numerical information: symbolic and nonsymbolic (Ansari 2008; Verguts and Fias 2008).

The symbolic system refers to our ability to use abstract numerical symbols (e.g., Arabic numerals) to perform exact calculations. This system is uniquely human, as it is related to language and culture (Merkley and Ansari 2016; O'Shaughnessy et al. 2021). However, few studies showed that chimpanzees can be trained to associate Arabic numerals with corresponding quantities and to arrange them in ascending order, suggesting that they possess a basic understanding of both cardinality (quantity) and ordinality (numerical order) (Matsuzawa 2009). Moreover, extensive research has demonstrated that Grey parrots could quantify sets of items using vocal English labels and comprehend their meaning (Pepperberg 2006), while crows have been shown to recognise Arabic numerals and produce a matching number of actions (e.g., pecks or vocalisations) (Kirschhock and Nieder 2023; Liao et al. 2024). Despite these findings, the ability of non‐human species to understand and use Arabic numerals appears to depend on extensive and specialised training.

In addition to the symbolic system, people are also equipped with a nonsymbolic numerical system for representing and manipulating numerical quantities (i.e., the number of items in a set, here referred to as ‘numerosity’) without true counting or numerical symbols (Dehaene 2011). This intuitive sense of number is not unique to humans but is found in a wide range of species, from bees to primates. The shared ability to discriminate between numerosities underscores the critical role that number sense has played in survival and adaptation throughout evolution (Boysen and Capaldi 2014; Nieder 2020; Butterworth 2022).

Two distinct but interrelated representational systems underlie nonsymbolic numerical cognition: the object tracking system (OTS) and the approximate number system (ANS) (Feigenson et al. 2002, 2004; Hyde 2011; Piffer et al. 2012; Szabo et al. 2021). The OTS functions as an attention‐based mechanism that creates specific memory entries for each perceived item in the environment. This system allows for rapid and precise representation of small numbers of objects, typically up to 4. The ANS, on the other hand, enables imprecise estimation of large numerosities (larger than 4) in a ratio‐dependent manner, consistent with Weber's law, according to which the just‐noticeable difference between two stimuli is proportional to the magnitude of the stimuli. Although the OTS and ANS are traditionally distinguished by numerical range, evidence of ratio‐dependent effects in small‐number discriminations suggests that ANS‐like mechanisms can also operate below large numerosities (Starr et al. 2013; Potrich et al. 2015; Ditz and Nieder 2016; Bengochea et al. 2023).

Evidence linking performance in nonsymbolic numerical tasks (e.g., judging which of two dot arrays is numerically larger) to mathematical achievement (Halberda et al. 2008; Schneider et al. 2017; Fyfe et al. 2019), together with findings showing that training in nonsymbolic numerical processing enhances symbolic arithmetic abilities (Hyde et al. 2014; Park and Brannon 2014), suggests that symbolic mathematics is grounded in nonsymbolic representations of numbers. Beyond the theoretical importance, this relationship has significant translational implications for understanding disorders characterised by atypical numerical processing, such as developmental dyscalculia, a specific learning disorder affecting mathematical skills (Butterworth 2018) characterised by deficits in both symbolic and nonsymbolic numerical abilities (Decarli et al. 2023; Dolfi et al. 2024). Research has also revealed intriguing links between numerosity skills and neurodevelopmental disorders such as Williams–Beuren syndrome (WBS) (Van Herwegen et al. 2008; O'Hearn et al. 2011) and Fragile X syndrome (Murphy et al. 2006; Murphy and Mazzocco 2008), both of which have well‐defined genetic origins.

Research on atypical developmental trajectories and genetic disorders linked to numerical impairments provides valuable insights into the processes that might be involved. However, studies in humans offer only a partial view of the mechanisms underlying numerical cognition. In this context, animal models could serve as a powerful tool to investigate the biological and neural bases of numerical abilities, allowing controlled manipulations of genes, brain circuits and early environmental factors that are not feasible in human studies. Additionally, they enable the study of numerical cognition development under highly controlled conditions, minimising the influence of cultural factors such as language and education. Together, cross‐species evidence from behaviour, neural activity and genetics can significantly deepen our understanding of the origins of numerical competence.

Building on this perspective, we adopt an integrative approach to numerical cognition, bringing together evidence from behavioural, genetic and neural levels. We first outline behavioural findings across species, showing how animals perceive and respond to numerosity in natural contexts and how these abilities can be studied under controlled laboratory conditions. Next, we explore the genetic basis of number sense, drawing on both human studies and research in zebrafish, a well‐established model in translational cognitive neuroscience. Finally, we highlight recent discoveries about the neural mechanisms that support numerical processing in non‐human animals. Overall, we offer a cohesive framework for understanding both the biological roots and the evolutionary development of numerical abilities.

This manuscript synthesises key themes and recent advances discussed at the 20th meeting of the Spanish Society for Neuroscience (SENC) celebrated in Las Palmas de Gran Canaria, September 2025, on the neural substrates of number sense. Where relevant, we highlight illustrative examples from our own recent work alongside established findings from the broader literature. By integrating behavioural, genetic and neurobiological perspectives across species, we aim to outline a framework for understanding how numerical cognition emerges from conserved biological mechanisms. This integrative perspective may also help identify biologically grounded markers of importance to address mathematical learning difficulties.

## 2 Developmental Perspectives on Numerical Cognition

The widespread occurrence of numerical abilities in both vertebrates and invertebrates indicates that numerical competence is adaptive, conferring advantages in several ecological contexts such as social interactions, foraging, hunting, predation avoidance and mate choice (Nieder 2020). For instance, in chimpanzees, hyenas and lionesses, decisions to attack rival groups are largely driven by numerical advantage, which reduces the risk of costly defeats (Benson‐Amram et al. 2018). Optimal group size is also fundamental for predatory animals, increasing hunting success in species as different as wolves and spider‐eating spiders ( *Portia africana* ) (MacNulty et al. 2014; Barber‐Meyer et al. 2016). Similarly, male frogs of different species match or exceed the number of calls produced by competitors to attract females (Rose 2018).

### 2.1 Balancing Ecological Relevance and Experimental Control in Numerical Cognition

Field studies are crucial for shedding light on the role of numerical abilities in natural environments but are limited by difficulties in accessing species and controlling continuous non‐numerical variables that covary with numerosity (e.g., volume, brightness and density), which animals may use instead of number per se to discriminate between quantities (Leibovich et al. 2017). These problems can be by‐passed in laboratory studies using two main methodological approaches: spontaneous choice tests and training procedures (Agrillo and Bisazza 2014).

In the former, untrained animals spontaneously discriminate between biologically relevant stimuli (e.g., food items and number of conspecifics) differing in numerosity under semi‐naturalistic conditions, supporting the ecological validity of numerical competence. For instance, several studies have exploited the spontaneous tendency of different fish species to join the larger between two shoals when exploring a novel environment, thereby reducing predation risk, to assess the limits of their numerical abilities (e.g., Agrillo et al. 2008; Gómez‐Laplaza and Gerlai 2011; Mehlis et al. 2015; Lucon‐Xiccato et al. 2017). In addition, vertebrates and invertebrates maximise their energy intake by selecting the larger amount of food (Bogale et al. 2014; Miletto Petrazzini and Wynne 2016; Yang and Chiao 2016; Gazzola et al. 2018; Schaffer et al. 2025) or the optimal quantity of dangerous prey (Panteleeva et al. 2013). Despite this ecological relevance, controlling continuous variables remains challenging when dealing with biologically salient stimuli. For instance, animals may prefer a larger food item that is easier to handle rather than a more numerous food option.

To overcome this issue, researchers have widely adopted training procedures where animals are trained to learn a numerical rule using neutral stimuli (e.g., two‐dimensional geometric figures), allowing strict control of continuous variables (see, for example, Zanon et al. 2022). Using this approach, animals can be trained to select specific numerosities (crows, rhesus macaques [Nieder 2018], guppies [Miletto Petrazzini et al. 2015], archerfish [Potrich et al. 2022] and honeybees [Howard et al. 2018]), process ordinal information (brown capuchin monkeys [Judge et al. 2005], honeybees [Dacke and Srinivasan 2008], pigeons [Scarf et al. 2011] and guppies [Miletto Petrazzini et al. 2015]) and to perform simple arithmetic operations (rhesus macaques [Cantlon and Brannon 2007] and honeybees [Howard et al. 2019]). Although training procedures are fundamental for demonstrating that animals can use numerical information, they are time‐consuming and lack ecological validity.

Nonetheless, it is worth noting that most studies are laboratory‐based and rely on captive‐bred individuals, which can limit how broadly the results apply to wild populations and may affect our understanding of true numerical competence. Captivity can alter cognitive performance (Baldoni et al. 2025), while the rearing environment can influence neural development and brain plasticity (Salvanes et al. 2013). For these reasons, it is necessary to integrate ecological context into cognitive research to understand the evolution and function of numerical abilities. In this light, spontaneous choice tests and training procedures should not be seen as alternatives, but rather as complementary approaches that together offer a more complete understanding of numerical competence in animals (Agrillo and Bisazza 2014; Nieder 2020). Although this concern has been highlighted mainly in fish, it clearly extends to other non‐human animals as well (Salena et al. 2021).

### 2.2 Evolutionary Origins of Numerical Competence: Homology or Convergence?

Numerical competence is a phylogenetically ancient ability that humans share with animals, yet its evolutionary origin remains debated. Two main hypotheses have been proposed. One possibility is that rudimentary numerical abilities are homologous traits inherited from a common ancestor dating back hundreds of millions of years (Nieder 2021). Alternatively, such abilities may have evolved independently through convergent evolution due to similar selective pressures. Despite broad continuity in numerical abilities across species, important anatomical and physiological differences in the telencephalic pallium, the brain area involved in numerical representations in birds, mammals and fish, support the hypothesis of an independent origin of numerical competence across taxa (Nieder 2021).

Finally, from a developmental perspective, numerical abilities emerge early in life, as newborns can discriminate between sets of objects with a 1:3 ratio (Izard et al. 2009), and then continue to develop until adulthood (Halberda and Feigenson 2008; Halberda et al. 2012). This developmental trajectory is mirrored across species, as comparable abilities have been documented in juvenile animals such as guppies, tadpoles, dogs, zebrafish and chicks (Balestrieri et al. 2019; Miletto Petrazzini et al. 2020; Sheardown et al. 2022; Adam et al. 2024; Lorenzi et al. 2025), indicating that numerical competence does not require extensive learning or experience and highlighting its crucial role for individual survival across species and developmental stages.

## 3 The Heritability of Numerosity: Findings in Human Studies

Although behavioural conservation suggests there is a genetic basis of number sense that may be conserved across species, analyses from human studies indicate that number sense shows only moderate heritability of around 30% (Tosto et al. 2014). Thus, accordingly, around 30% of the variance in number sense ability is predicted to be explained by genetic factors. This is consistent with findings for individual differences in almost all other human behaviours and cognition (Docherty et al. 2010; Briley and Tucker‐Drob 2017). Moreover, phenotypic variation in number sense may also reflect genotype‐environment interactions and experience‐dependent plasticity, suggesting that environmental factors can modulate the expression of genetically influenced numerical abilities.

Building on population genetic studies in animals and humans, Tosto et al. (2014) argued that the reduced heritability is the result of number sense being crucial for survival and thus subject to directional selection pressure over evolution (see Box 1): Directional selection reduces additive negative genetic variance, thus traits subjected to selection pressure would be expected to show lower heritability (Tosto et al. 2014). As a result of such selection pressure, genetic variants in existing healthy populations would be expected to have small effect to explain individual differences in ability. However, number sense may also be subject to indirect selection (see Box 1) that can lead to genetic variation associated with a specific trait being of small effect (Lande and Arnold 1983). In either case, identifying candidate genes from human GWAS (see Box 1) would be problematic, as each individual variant is likely to have a small effect resulting in GWAS having low power to detect the loci involved. This may explain why relatively few GWAS studies have focused on number sense. Nonetheless, the observation that number sense predicts mathematical ability suggests that genetic variants associated with mathematical ability may also influence number sense ability.

Box 1 **Genetic concepts relevant to the heritability of number sense.**

**Directional selection**: A form of natural selection in which individuals with trait values at one extreme of a distribution have higher fitness than others.

**Indirect selection:** A form of selection whereby changes in the frequency of an allele occur not because of its own effect on fitness (*direct selection*), but because it is genetically linked to another allele that is under direct selection. Mechanisms by which indirect selection may lead to variants of small effect include:

- *Linkage disequilibrium*: A genetic variant shows an association with a trait because it is close on the chromosome to a different variant that affects the trait.
- *Pleiotropy*: If a gene affects multiple traits, selection may act on it primarily through a different trait. Its effect on the trait of interest therefore appears weak as its population frequency reflects selection elsewhere.
- *Stabilising selection*: It removes large‐effect variants over time, as large effects risk pushing individuals away from the optimal trait value. This leaves a landscape dominated by many small‐effect variants.
- *Correlated trait selection*: A variant's apparent effect on a trait may simply be a statistical shadow of selection acting on a different correlated trait.

**Additive genetic variance**: The component of genetic variance attributable to the cumulative effects of individual alleles across loci. This component determines the extent to which genetic differences between individuals contribute to the heritable variation of a trait within a population.

**Heritability**: The proportion of phenotypic variation in a population that can be attributed to genetic differences among individuals. Heritability depends on both genetic variance and environmental influences and does not indicate the extent to which a trait is genetically determined in an individual.

**Polygenic architecture**: A genetic architecture in which a trait is influenced by many genetic variants, each typically contributing a small effect.

**Genome‐wide association study (GWAS)**: A statistical approach used to identify associations between genetic variants across the genome and variation in a trait within a population.

**Copy number variation (CNV)**: A type of structural genetic variation in which a segment of DNA is present in different numbers of copies across individuals.

Human GWAS for mathematical ability have reported significant association of variants in several genes (see Table 1 for list of genes and relevant references). In addition, a CNV (see Box 1) in chromosome 15q11.2 that disrupts *CYFIP1*, *NIPA2* and *NIPA1* is associated with dyscalculia (Table 1). Finally, genes involved in syndromes with a dyscalculia component, such as Fragile X, Prader–Willi, WBS and Turner's syndromes (Table 1), may also be candidate genes for the development and/or function of neuronal circuits underlying number sense.

**TABLE 1:** Candidate genes implicated in numerical cognition and mathematical learning difficulties.

Gene	Link to numerosity	Function	Shared process(es) affected	Empirical evidence of role in number sense established?	References
*ARID1B*	Identified as significant hit in GWAS for mathematics.	*ARID1B* encodes AT‐rich‐interactive‐domain‐containing protein 1B, a subunit of the BAF chromatin remodelling complex and, plays an essential role in neurite outgrowth and maintenance. In addition, *Arid1b* haploinsufficiency results in excitatory/inhibitory imbalance via decreased GABAergic interneuron numbers and impaired synaptic transmission of inhibitory signals.	Regulation of neural cell fate and maturation. Structural remodelling needed for neural circuit formation. Neural activity levels.	No	(Docherty et al. 2010; Ka et al. 2016; Jung et al. 2017; Moffat et al. 2019)
*CYFIP1/2*	1 of 4 genes in 15q11.2 (BP1‐BP2) associated with dyscalculia.	CYFIP1 and 2 (cytoplasmic FMR1 interacting protein) interact with FMR1 (Fragile X Mental Retardation) as part of the CYFIP1‐FMR1‐eIF4E pathway that regulates transcription. Through interaction with RAC1, CYFIP1 activates the WAVE regulatory complex and promotes cytoskeletal remodelling, a key aspect of dendritic spine formation. Altered *CYFIP1* dosage perturbs neuronal morphology, synaptic excitation and brain patterning and function.	Neural activity levels. Activity‐dependent strengthening and refinement of synapses. Structural remodelling needed for neural circuit formation.	No	(DeRubeis et al. 2013; Stefansson et al. 2014; Oguro‐Ando et al. 2015; Kendall et al. 2017; Davenport et al. 2019)
*DNAH5*	Identified as significant hit in GWAS for mathematics.	*DNAH5* (Dynein axonemal heavy chain 5) encodes an axonemal heavy chain dynein, which is part of a microtubule‐associated motor protein complex. Mutations in *DNAH5* cause ciliary defects leading to cortical development disorders and congenital hydroencephalus.	Structural remodelling needed for neural circuit formation.	No	(Docherty et al. 2010; Sakamoto et al. 2024)
*FMR1*	Fragile X Mental Retardation 1 (FMR1) pre‐mutations are associated with selective number processing deficits in individuals of normal intelligence FMR1.	FMR1 binds to CYFIP1 and 2 to regulate translation and actin dynamics.	Neural activity levels. Activity‐dependent strengthening and refinement of synapses. Structural remodelling needed for neural circuit formation.	No	(Loesch et al. 2003; DeRubeis et al. 2013)
*FZD9*	One of the genes deleted in WBS. Individuals with WBS show deficits in numerical approximation.	Fzd9 (Frizzled 9) encodes a Wnt receptor that regulates dendritic spine formation, axonal growth and synaptic maturation. Its loss is associated with altered neuronal morphology and learning and memory deficits. FZD9 is required for normal cortical neuron development, likely via modulation of excitatory‐inhibitory balance.	Neural activity levels. Activity‐dependent strengthening and refinement of synapses.	Yes	(Rousselle et al. 2013; Libertus et al. 2014; Chailangkarn et al. 2016; Ramírez et al. 2016)
*GRIK1*	Identified as significant hit in GWAS for mathematical ability.	GRIK1 (glutamate receptor ionotropic kainate) encodes kainate ionotropic glutamate receptor subunit 1 (KAR1). KARs coordinate and regulate neuronal and network activity via regulation of both excitatory and inhibitory transmission.	Neural activity levels (balance between excitatory and inhibitory signals). Activity‐dependent strengthening and refinement of synapses.	No	(Docherty et al. 2010; Baron‐Cohen et al. 2014; Negrete‐Díaz et al. 2022)
*GUCY1A2*	Identified as significant hit in GWAS for mathematical ability.	GUCY1A2 (guanylate cyclase soluble subunit alpha‐2) encodes one of the alpha subunits of the soluble guanylate cyclase, a ubiquitously expressed enzyme that is the only known receptor for nitric oxide. Nitric oxide signalling modulates synaptic strength.	Activity‐dependent strengthening and refinement of synapses.	No	(Garthwaite 2008; Docherty et al. 2010)
*MMP7*	Identified as significant hit in GWAS for mathematics	MMP7 (matrix metalloproteinase‐7) is a member of a family of enzymes that can collectively cleave all extracellular matrix (ECM) components in addition to several cell surface proteins. MMP cleavage of ECM, membrane and pericellular proteins can result in complex changes to synaptic homeostasis and signalling; MMPs are likely to also play a role in synaptic development and remodelling.	Cell adhesion and extracellular matrix. Structural remodelling needed for neural circuit formation.	No	(Mott and Werb 2004; Docherty et al. 2010)
*MYO18B*	Identified as a significant hit in GWAS for mathematics in children with dyslexia.	*MYOSIN18B* encodes a member of the myosin family that lacks ATPase activity. It is expressed at low level in many cell types. Involved in actin–myosin interactions important for formation of stress fibres and cell migration.	Structural remodelling needed for neural circuit formation.	No	(Ludwig et al. 2013; Ouyang et al. 2021)
*NIPA1/2*	2 of 4 genes in 15q11.2 (BP1‐BP2) associated with dyscalculia.	NIPA1 and 2 (nonimprinted in Prader–Willi/Angelman) encode Mg^2+^ transporter proteins. Mg^2+^ levels regulate circuit formation during brain development and play a role in both GABA and glutamate signalling.	Structural remodelling needed for neural circuit formation. Activity‐dependent strengthening and refinement of synapses. Neural activity levels.	No	(Goytain et al. 2007, 2008; Stefansson et al. 2014; Kendall et al. 2017; Yamanaka et al. 2018, 2019)
*NRCAM*	Identified as significant hit in GWAS for mathematical ability. Associated with autism.	NRCAM (neural cell adhesion molecule) is important for synaptic adhesion and stabilisation.	Activity‐dependent strengthening and refinement of synapses. Structural remodelling needed for neural circuit formation. Cell adhesion and ECM interactions.	No	(Hutcheson et al. 2004; Docherty et al. 2010; Duncan et al. 2021)
*NUAK1*	Identified as significant hit in GWAS for mathematics.	NUAK1 encodes AMPK‐related protein kinase 5 (thus, also known as ARK5). NUAK 1 and 2 are important for establishing neuronal shape that is crucial for building functional circuitry in the brain. Nuak1 has been implicated in neurite formation as well as terminal axon and dendritic branching through regulation of actin contractility, mitochondrial transport and microtubules.	Structural remodelling needed for neural circuit formation.	No	(Docherty et al. 2010; Bennison et al. 2022)
*SMAD3*	Identified as significant hit in GWAS for mathematics.	SMAD3 is a member of the suppressor of mother against decapentaplegic family of proteins. SMAD3 is one of the principal transducers of signals from TGF‐β receptors. TGF‐β signalling influences neural differentiation and maturation. Neuronal and astroglial TGFβ‐Smad3 signalling pathways differentially regulate dendrite growth and synaptogenesis.	Regulation of neural cell fate and maturation. Structural remodelling needed for neural circuit formation. Activity‐dependent strengthening and refinement of synapses.	No	(Docherty et al. 2010; Wang and Symes 2010; Yu et al. 2014)
*SPOCK1*	Identified in GWAS for mathematics ability.	SPOCK1 (SPARC/osteonectin, CWCV and Kazal‐like domains proteoglycan 1) is a highly conserved, multi‐domain proteoglycan that regulates the dynamic equilibrium of extracellular matrix.	Cell adhesion and ECM composition. Structural remodelling needed for neural circuit formation.	No	(Bradshaw 2012; Chen et al. 2017)

While these analyses suggest the presence of identifiable genetic underpinnings of numerical cognition, to date, there is no clear genetic or molecular understanding of how genetic risk factors might contribute to defects in number processing. However, the abovementioned genes are all predicted to regulate neuronal connectivity either directly or indirectly during development (see Table 1 for potential mechanisms). Although empirical evidence for a role in number sense is missing for all but one of the listed genes, interestingly, several of the gene products regulate the balance between inhibitory and excitatory signalling in the brain and/or directly affect glutamatergic signalling and synapse formation. This may be of relevance as computational models of how we extract spatial information from a visual scene, which are necessary to identify the number of items in the scene, are often based on the concept of a ‘saliency map’, the generation of which relies on a balance between excitatory and inhibitory signals (Roggeman et al. 2010; Sengupta et al. 2014; see below).

Thus, the concept of a saliency map provides a framework linking neural circuit dynamics to numerosity perception. As several candidate genes discussed above influence excitatory/inhibitory balance and synaptic organisation, these models provide a useful conceptual bridge between genetic variation and numerical cognition.

## 4 The Role of Saliency Maps in Number Cognition

In computational models of numerosity perception, number extraction is thought to operate on the principle of a ‘saliency map’, where individual objects generate discrete activity peaks or nodes that compete with their neighbours for neural representation.

These nodes follow two crucial rules. First, self‐excitation means that when a node detects something, it boosts its own signal. This self‐reinforcement helps the brain lock onto and maintain focus on detected objects. Second, lateral inhibition means each active node tries to suppress the activity of neighbouring nodes around it. When one location is being actively processed, nearby locations get temporarily suppressed so they do not interfere. Together, these rules create a competitive interaction map. Only the most salient items ‘win’ this competition and become the focus of conscious awareness. This competition is hypothesised to be crucial for counting and assessing numerosity because it allows significant items to be extracted from background information, preventing the brain from being overwhelmed by processing everything simultaneously.

This model suggests that variations in excitatory and inhibitory signalling will significantly influence the ability to discriminate number. The strength of excitatory signalling would determine how strongly nodes respond, with objects that create stronger signals being easier to detect. The strength of inhibitory signalling would determine how much neighbouring nodes get quieted, that is, stronger inhibition creates sharper competition, making it easier to focus on individual objects but potentially harder to detect closely spaced items.

The practical implications are significant: If the balance between excitation and inhibition is disrupted, perhaps due to genetic differences, neurological conditions or temporary states like fatigue, this could explain why some individuals struggle with tasks like quickly estimating quantities or counting objects in cluttered scenes. The brain's counting ability thus depends on a delicate competitive balance between excitatory and inhibitory signalling where different parts of the visual system vie for attention. These hypotheses can be tested in animal models, especially those amenable to genetic manipulation. Although this framework provides a plausible mechanistic link between neural circuit properties and numerosity perception, the extent to which these processes directly mediate numerical cognition remains to be tested experimentally.

## 5 Zebrafish ( *Danio rerio* ) as an Animal Model for Numerosity

Relatively few studies have used animal models to investigate the role of specific genes in numerical cognition. One major limitation has been that, until recently, neural correlates of number sense had not been identified in genetically tractable species such as mice, zebrafish or *Drosophila*. However, the recent identification of a specific population of neurons required for behavioural responses to number in *Drosophila* (Bengochea et al. 2023), together with preliminary data from our group identifying number‐responsive neurons in zebrafish (discussed later), indicates that these species provide experimentally tractable systems in which to address genetic mechanisms underlying number sense.

To capitalise on these advantages, we developed a confined‐stimulus group size preference (GSP) assay to assess quantity discrimination in juvenile zebrafish (Sheardown et al. 2022). This zebrafish assay, based on an approach previously developed by Gómez‐Laplaza and Gerlai (2012) using angelfish, exploits the spontaneous tendency of fish to associate with the larger of two shoals when placed in a novel environment, a behaviour linked to reduced predation risk. Individual fish explore a central arena while viewing two groups of conspecifics presented behind transparent barriers that differ in group size, with preference measured as time spent near the larger group. The task requires no training and relies on spontaneous behaviour, combining experimental control with ecological relevance (see section above).

In our previous work using juvenile zebrafish (Sheardown et al. 2022), we demonstrated that individuals reliably discriminate large‐quantity differences (e.g., 2 vs. 5) but fail at intermediate contrasts (e.g., 2 vs. 4) despite identical ratios. This pattern leads us to propose that quantity discrimination may be constrained by cognitive processes involved in tracking and comparing multiple items. Particularly, the OTS relies on attentional resources to monitor discrete objects, while working memory is required to temporarily maintain the information related to those items during the comparison paradigm. Therefore, when the number of individuals being tracked exceeds these attentional or working‐memory capacities, the discrimination performance of the fish deteriorates, even when numerical ratios remain favourable. Based on these findings, we proposed that this paradigm provides a useful behavioural framework for probing how genetic manipulations affect numerosity‐based decision making.

A key advantage of these animal models is the ability to perform whole‐brain imaging in vivo while animals engage in numerical stimuli observation, enabling direct links to be drawn between gene function, neural circuit activity and behaviour. In this context, our recent work (see below) further supports the translational relevance of zebrafish for studying the genetic and neural bases of numerical cognition.

### 5.1 WBS: Insights From Zebrafish Models

WBS (also known as Williams syndrome; OMIM: 194050) is a rare neurodevelopmental disorder caused by a hemizygous microdeletion on chromosome 7q11.23, typically spanning 1.55–1.8 Mb and encompassing around 25–28 genes. In humans, this deletion is the only known cause of the syndrome and directly affects how the cognitive, behavioural and neurobiological traits are observed in the affected individuals of both sexes.

WBS is associated with congenital heart malformations as well as hypertension, endocrine abnormalities, short stature and hyperacusis (Pires et al. 2021). Interestingly, from a neuropsychological standpoint, WBS is highly heterogeneous. People with WBS have often been described as showing mild intellectual disability (Kozel et al. 2021), yet some studies report that up to half of individuals can score within the typical IQ range (Pitts and Mervis 2016; Hsu and Jiang 2025). Regardless of IQ range, individuals with WBS tend to share a recognisable profile: hypersociability, anxiety proneness, strong interest in music and impairments in motor, executive and visuospatial abilities, as well as difficulties with grammar and using language appropriately in conversation (syntactic and pragmatic‐discursive abilities), while vocabulary knowledge is relatively well preserved (Carvalho and Haase 2019; Kozel et al. 2021). This combination of cognitive domains, with some affected and others spared, has led WBS to be considered a prototypical cause of non‐verbal learning disability, where problems with visuospatial and executive skills stand out against relatively strong verbal skills (Bellugi et al. 2000; Martens et al. 2008).

Consequently, WBS has been widely recognised as a model for studying how genetics affects mathematical learning. Individuals with WBS show impaired subitising, reduced nonsymbolic numerosity accuracy for small and medium sets and difficulties with number‐line and magnitude comparison tasks, although symbolic processing may be relatively spared (Van Herwegen et al. 2008; O'Hearn and Luna 2009; O'Hearn et al. 2011; Ranzato et al. 2020; Simms et al. 2020). These patterns are consistent with previous evidence of reduced ANS precision, a narrower subitising range and atypical developmental trajectories for numerical and spatial processing, while time processing appears comparatively less affected (Libertus et al. 2014; Van Herwegen et al. 2020). Taken together, they support the view that WBS is characterised by a selective, early disruption of core numerical and visuospatial systems, which in turn contributes to persistent mathematical learning difficulties.

We have taken advantage of the capabilities discussed above of zebrafish and our GSP assay to probe how individual WBS‐associated genes contribute to early quantity processing. In that study, we tested juvenile zebrafish (30–35 days postfertilisation, dpf) carrying loss‐of‐function (LoF, generated using the CRISPR/Cas9 system) mutations in *baz1b* or *fzd9b*, two of the genes with strong links to WBS neurodevelopmental phenotypes (Torres‐Pérez et al. 2026).

*BAZ1B* encodes a chromatin remodeller involved in neural crest development, craniofacial morphology and social behaviour, as demonstrated in both mice and humans (Sun et al. 2016; Zanella et al. 2019; Pai et al. 2024). In our zebrafish LoF line, reduced *baz1b* expression leads to mild neurocristopathy, distinctive craniofacial changes and a combination of reduced stress reactivity and altered social ontogeny, mirroring domestication‐like phenotypes (Torres‐Pérez et al. 2023). Together, these findings support a central role for *BAZ1B* in coordinating neural crest‐derived morphology and prosocial behaviours, in line with its proposed contribution to WBS.

Similarly, *fzd9b* encodes Frizzled 9b, a Wnt receptor involved in neurodevelopment, dendritic spine formation and axonal growth, with loss of *FZD9* previously linked to aberrant neuronal morphology and learning and memory deficits in mammalian models (Ramírez et al. 2016; Pascual‐Vargas and Salinas 2021). In our zebrafish LoF lines, disrupting *fzd9b* altered stress‐ and anxiety‐related behaviours at both larval and adult stages (Torres‐Perez et al., n.d.). Thus, our *fzd9b* findings support a key role for Fzd9b in regulating stress reactivity and anxiety phenotypes relevant to WBS.

Once they were characterised, we assessed both LoF lines in our confined‐stimulus GSP assay across three quantitative contrasts (2 vs. 5, 2 vs. 4 and 2 vs. 3). Importantly, on this occasion, we framed the assay in terms of ‘quantity’ rather than ‘number’, because we did not control for overall area or other continuous variables that we had manipulated in previous work (Sheardown et al. 2022). Therefore, fish could base their choices on both discrete numerosity (count of conspecifics) and continuous variables (e.g., overall area and movement). Treating these as expressions of a common magnitude system allowed us to assess the contribution of WBS genes to quantity discrimination in a way that more closely reflects the mixed discrete/continuous cues also present in human studies of numerical cognition.

In the *baz1b* LoF, wild‐type (WT) and heterozygous fish showed a significant preference for the larger shoal in the easiest contrast (2 vs. 5), whereas homozygous mutants did not, while no genotype showed a clear preference in the more difficult contrasts (2 vs. 4 and 2 vs. 3). In the *fzd9b* line, WT fish preferred the larger shoal in both 2 vs. 5 and 2 vs. 4, but not 2 vs. 3. Strikingly, heterozygous *fzd9b* fish showed the opposite pattern, succeeding only in the most difficult contrast (2 vs. 3), while homozygous mutants failed to show a reliable preference in any condition. Consistent with our previous work, where performance drops when the total number of items exceeds about five (a putative working‐memory limit; see above), these patterns suggest that LoF in *baz1b* and *fzd9b* selectively impairs large‐quantity processing, which is more likely to recruit ANS‐like mechanisms, while small‐quantity processing through an OTS‐like system seems mostly spared.

These data provide preliminary but convergent evidence that *BAZ1B* and *FZD9* contribute to the nonsymbolic quantity‐processing deficits observed in WBS, particularly for larger sets where approximate representations and ratio sensitivity are critical. The selective impairment in large‐quantity discrimination is consistent with findings in individuals with WBS (Van Herwegen et al. 2008; Rousselle et al. 2013; Libertus et al. 2014). Therefore, our study strengthens the case for zebrafish as a translational model to dissect the genetic and neural bases of dyscalculia in WBS and related conditions. Overall, these data begin to clarify how these genes shape behavioural quantity discrimination, highlighting the need to resolve the underlying molecular and circuit mechanisms.

## 6 Neural Correlates of Numerosity Across Development

As discussed above, behavioural evidence across species indicates that the ability to perceive and estimate numerical quantity is a core cognitive faculty (e.g., Hersh and Dehaene 1998; Lipton and Spelke 2003; Piffer et al. 2013; Howard et al. 2019; Bortot et al. 2021; Messina, Potrich, Perrino, et al. 2022; Sheardown et al. 2022; Zanon et al. 2025). We have also reviewed the initial efforts to determine the genetic characterisation of this number sense in zebrafish. Here, we will focus on the neuronal and circuit mechanisms underpinning it.

Preliminary studies identified specific brain areas maximally involved during numerical cognition tasks, in particular pallial regions in the zebrafish brain (Messina et al. 2020; Messina, Potrich, Schiona, et al. 2022). Interestingly, these findings seem conserved across species, with analogous evidence in different animal models (see, e.g., Piazza et al. 2004; Nieder 2018; Lorenzi et al. 2021, 2024; Kobylkov et al. 2023).

At the level of single neuronal units, there is also large evidence for specialised cells encoding numerical information. While some studies emphasise the existence of ‘monotonic neurons’, whose responses increase or decrease monotonically with perceived numerosity (Roitman et al. 2007), accumulating evidence indicates that numerical perception signatures are well described by specialised populations of ‘number neurons’, cells that respond preferentially to specific number of elements (i.e., numerosities) and whose activity decreases as the numerical distance from the preferred value increases (tuned responses consistent with Weber's law; Nieder 2016; see, Box 2).

Box 2 **Key concepts in neural coding and decoding.**

**Tuning curve**: The relationship between neuronal activity and a stimulus feature. In numerosity‐selective neurons, it describes how firing rate varies as a function of numerosity, typically showing a peak (preferred numerosity) and graded responses to nearby values. This specific behaviour usually emerges more clearly as a population coding more than at single neuron level.

**Population coding**: A form of neural representation in which information about a stimulus is encoded by the combined activity of a population of neurons, rather than by single cells carrying the full information.

**Neural decoding**: A computational approach used to determine whether patterns of neural activity contain information about a stimulus or behavioural variable. Typically, this is achieved by training a model (e.g., an SVM) to predict those variables from the recorded neuronal activity.

**Support vector machine (SVM)**: A supervised machine learning classifier. It can be trained to associate patterns of neuronal activity with stimulus categories and then tested on new data to evaluate whether the neural population reliably encodes those categories.

In mammals, number neurons have been identified in parietal and prefrontal cortices (Nieder et al. 2002; Roitman et al. 2012), and similar cells have been detected in the Nidopallium Caudolaterale (NCL), an integrative area analogous to the mammalian prefrontal cortex, in crows and chicks (Wagener et al. 2018; Kobylkov et al. 2022). These neurons collectively form a distributed code capable of supporting the discrimination, comparison and manipulation of numerical quantities, providing a framework for understanding how numerical cognition emerges from neural circuits.

Despite these advances, how these neural correlates emerge during development remains poorly understood, particularly in genetically tractable vertebrates such as zebrafish.

While numerosity‐selective neurons have previously been reported in mammals and birds, our recent study using functional calcium imaging revealed such neurons in larval zebrafish (3–7 dpf), providing one of the earliest cellular correlates of approximate numerosity coding in this vertebrate model (Luu et al. 2026). In this study, neurons whose activity varied systematically with the number of items presented (1–5 black dots on a red background) were identified across the whole larval brain. We can describe these responses as consistent with ANS, because the paradigm used nonsymbolic dot displays, revealing Weber‐consistent tuning curves and ratio dependence across numerosities. Nonetheless, we acknowledge that small‐number processing can recruit additional mechanisms (including OTS attention‐based tracking), and future work will be needed to formally dissociate these contributions.

Moreover, the informational content of these neurons was quantified by applying machine learning decoding analyses using a support vector machine (SVM) classifier (see Box 2). This is a decoding algorithm that assigns neuronal activity patterns to stimulus categories, predicting the numerosity viewed by the animal from the recorded ‘number neurons’ population activity. This demonstrates that the neuronal ensemble contains sufficient discriminative information to encode the number of elements with high fidelity. Notably, decoding remained robust even with relatively small subsets of neurons and could be generalised across fish, indicating that numerical information is distributed across multiple neurons while retaining a structured, decodable population code which is shared across individuals. Together, these findings support the idea that the fundamental principles of numerical representation are conserved across vertebrates and are represented at the level of individual, identifiable neurons early in development, with zebrafish larvae exhibiting these properties already at 3 dpf.

Notably, this work provides initial evidence for a developmental trajectory of numerical representations, with early stages biased toward smaller numerosities and a later emergence of responses to higher quantities. As such, these findings open the possibility of directly investigating how numerical coding develops at the level of identified neurons, offering a tractable model to study the ontogeny and mechanisms of nonsymbolic number processing in vertebrates.

Overall, these results have broad implications for our understanding of the evolution and ontogeny of numerical cognition. They demonstrate that core aspects of the ANS are instantiated at the cellular level early in development and highlight zebrafish as a powerful system for linking neural circuit dynamics to cognitive functions. The identification of number‐selective neurons in a genetically tractable vertebrate model provides a platform for mechanistic interrogation of how numerical representations are formed and maintained. Future studies should aim to manipulate these circuits in vivo, for example, using targeted optogenetic or pharmacological perturbations (or, as discussed above, systematically testing genetically modified lines), to directly assess the causal role of number‐selective neurons in behaviour and to probe the neural bases of numerical deficits such as dyscalculia.

## 7 Conclusions and Future Directions

Number sense represents a core cognitive faculty that supports the ability to estimate and compare quantities without symbolic representations. The ubiquity of this ability across species highlights the adaptive value of numerical cognition. However, as we have argued throughout this review, understanding the biological basis of number sense requires integrating evidence across multiple levels of analysis. Therefore, progress depends on the convergence of cross‐species behavioural studies, developmental investigations, human and animal genetic analyses and circuit‐level approaches in experimentally tractable models.

This integrative perspective provides a framework for linking candidate genes, neural circuit dynamics and numerical behaviour, and for identifying conserved mechanisms that may underlie numerical cognition and its disruption. To bridge the gap between descriptive associations and mechanistic understanding, we identify in Box 3 some of the most relevant near‐ and long‐term objectives for the field.

Box 3 **Future perspectives and open questions.**

**Table:** 

Timeline	Research objectives and open questions
**Near‐term (empirical)**	**Gene‐to‐circuit mapping:** Systematic testing of candidate genes (e.g., from WBS and Fragile X) in tractable models (e.g., zebrafish and *Drosophila*) to identify specific effects on number‐selective neuron development.
	**Developmental windows:** Using whole‐brain imaging to pinpoint the exact developmental onset of the ANS vs. OTS and determine if early disruptions predict later cognitive deficits. Carefully map the developmental emergence of number‐selective neurons.
	**Causal manipulations:** Utilising optogenetics to activate/silence number‐selective neurons in zebrafish to dissect their role in numerical tasks.
**Long‐term (theoretical)**	**The symbolic bridge:** How are evolutionarily conserved, nonsymbolic circuits ontogenetically recycled or integrated with language‐based cortical networks to support the acquisition of abstract mathematical symbolism in humans?
	**Diagnostic biomarkers:** Can early neural signatures of numerosity or genetic risk scores in infancy serve as reliable predictive biomarkers for the later emergence of developmental dyscalculia?
	**Synthesising numerical systems:** Future research must move toward a unified model of numerical cognition that accounts for the interplay between the OTS, ANS and symbolic systems. This requires understanding the neural mechanisms that allow amodal representations of quantity to be mapped onto cultural symbols and how this synthesis matures from infancy through adulthood.

## Author Contributions

**Mirko Zanon:** visualization, writing – original draft, writing – review and editing. **Caroline H. Brennan:** visualization, writing – original draft, writing – review and editing. **Maria Elena Miletto Petrazzini:** visualization, writing – original draft, writing – review and editing. **Jose V. Torres‐Pérez:** conceptualization, visualization, writing – original draft, writing – review and editing.

## Funding

J.V.T‐P. is funded by the Spanish Ministry of Science, Innovation and Universities (MCIN/AEI/10.13039/501100011033) and the European Union ‘NextGenerationEU’/PRTR with a Ramón y Cajal contract (grant RYC2021‐034012‐I). J.V.T‐P. is also supported by the *Conselleria de Educación, Cultura, Universidades y Empleo* from the *Generalitat Valenciana* with a *Subvencion a grupos de investigación emergentes* (grant CIGE/2024/73).

## Conflicts of Interest

The authors declare no conflicts of interest.

## Data Availability

No new data have been generated in the present manuscript. For unprocessed dataset, refer to the corresponding author(s) from the original manuscript (see References list).
